# Mr. Wilson's Lecture on the Structure and Physiology of the Parts Composing the Skeleton

**Published:** 1820-12-01

**Authors:** 


*Lectures on the Structure and Physiology of the Parts composing the Skeleton, and on the Diseases of the Bones and Joints of the Human Body, preceded by some Observations on the Influence of the Brain and Nerves, delivered before the Royal College of Surgeons of London, in the Summer of the Year* 1820. By James Wilson, F. R. S. Professor of Anatomy and Surgery to the College, Lecturer of Anatomy and Surgery at the Hunterian School in Great Windmill Street, and one of the Vice Presidents of the Medico-Chirurgical Society of London. London, 1820. One vol. 8vo, pp. 416. Burgess & Co.

> "Ossa sub incurvis extabant arida Lumbis;

> " Auxerat articulos macies, Genuúmque tumebat

> " Orbis."

THIS work consists of fifteen lectures, and a short appendix. The first lecture is introductory, but contains many interesting observations on the brain and nervous system, in which Mr. Wilson takes an opportunity of passing a deserved eulogy on the erudite and valuable work of Dr. John Cooke.* The

* Treatise on Nervous Diseases.

2d lecture is "on the structure of bone, periosteum, and marrow;"—the 3d. "on the formation of bone, the structure of cartilage and ligaments, the properties of synovia, and the strength and motion of joints." The subject of the *fourth* lecture is the structure, formation, and time of appearance of the temporary and permanent teeth. The *fifth* lecture embraces general observations on the human skeleton. The *sixth* is on the situation, connexion, motion, &c. of the upper and lower extremities. The *seventh* on RICKETS, particularly as affecting the spine and pelvis. The *eighth* lecture is on inflammation, suppuration, and ulceration of bone, embracing simple fracture and subsequent union. The *ninth* is on the treatment of fractures, simple and compound. The *tenth* lecture is on fracture of the neck of the femur, mollities ossium, &c. &c. The *eleventh* is on exostosis, necrosis, and exfoliation of bone. The *twelfth* is on diseases of the joints. The *thirteenth* is on inflammation of the synovial membrane, &c. The *fourteenth* is on scrofula generally, and on scrofulous affections of joints. The *last* lecture is on abscesses of the hip-joint, and on incurvations of the spine from carious vertebræ. The *Appendix* is on the form and particular situation of the different classes of the adult teeth.

From this enumeration it will be sufficiently obvious that the work contains a great mass of important, elementary, and other matter, which it is impracticable to analyze, as Mr. Wilson's language is too didactic and precise to admit of much abbreviation. We have, on this account, determined to take a single lecture for analysis; as this will afford a specimen of the matter and manner of the publication, without leading us into that desultory patch-work species of medical reviewing, which grasps at much and does nothing—save blind the understanding by bewildering the sense. When, therefore, we cannot present a continuous and distinct view of a work, we shall generally select a portion of it, and do that portion justice. This, we imagine, will be found the most advantageous plan, ultimately, for every party concerned—and, at all events, it is indispensible to the spirit and design of this journal, which aims at other attractions than the splendor of variety and gloss of novelty to embellish the first few hours of an ephemeral existence.

We shall select the seventh lecture, the subject of which is RICKETS, for the present article.

We now know that bones have life, nerves, vessels, and nearly the same general structure as the soft parts of the body; "although their active powers of life, and extent of vascularity are less, on account of their requiring a greater degree of solidity, and stronger cohesion of their component parts."

As a large portion, however, of inert matter (phosphate of lime) enters into their composition, the performance of their internal actions must be more difficult and slow than in more highly organized structures:—the diseases of bones must therefore be necessarily of longer continuance. Mr. Wilson thinks that rickets arise from a deficiency of some of the materials which should enter into the composition of bone, since in this disease they lose their characteristic qualities of hardness and tenacity, bending under the weight which they ought to support, or becoming crooked by the effect of muscular action. This disposition shews itself at a very early period of existence—even in utero; but the more usual period of its occurrence is between the eighth month and the end of the second year. When it attacks after puberty, it is principally the spine, which suffers considerable lateral incurvation, more frequently happening in the female than in the male. Although often combined with scrofula, Mr. Wilson avers that its effects are distinct from those of the latter disease.

" The softening of the bones in rickets has been variously accounted for. It has been supposed by some Nosologists to arise from an excess of acid decomposing the phosphate of lime; by others, from the constitution not providing enough of this material; some have imagined that it is produced by the absorbent vessels having a morbid disposition to remove too much of this phosphate after it had been deposited in the bones; others have supposed it to originate from the arteries of the bones being deficient in the power of secreting phosphate of lime from the blood, and thus not depositing it in sufficient quantity in their substance. But we possess no proofs of excess of acid, or of any acid whatever being generated; for none has been found peculiar to rickety patients by any chemical, or other tests yet known. In rickety constitutions there does not appear to be any deficiency of the phosphate of lime in the blood generally; for in incurvations of the spine a redundancy of osseous matter is often pressed out on the bent side. This matter in rickety persons, is sometimes deposited in large quantities in parts not intended to be bony or even hard; and the urine of such persons is often found to be highly saturated with the phosphate of lime; moreover the absorbents do not remove it when deposited on the surface of the bones, or in parts external to them and unconnected with them. All these circumstances give greater probability to the opinion which presumes that the arteries of bones are deficient in the power of separating the phosphate of lime from the blood, so as to deposit it in sufficient quantity in their substance, than that any deficiency of this material exists in the body." 164.

In children of an early age there are certain *constitutional* indicia of the presence of rickets; but in later periods of life the disease is principally marked by the local affections it produces. These constitutional indicia are—diminished digestive powers, while the appetite is often voracious—swelled abdomen—flatulency—emaciation—relaxed bowels. The countenance, however, is lively, the eyes bright—and the intellectual powers precocious: probably from the inactivity of the animal functions, and association with seniors.*

"As the disease proceeds, the skin becomes dry and scaly, the teeth become black and decayed; the weight of the body cannot be supported in any position without producing curvatures of the bones; the natural functions of the internal organs are interrupted, the lungs become tuberculated and consumptive, and hectic symptoms arise, which are terminated by death." 166.

Nature often makes an effort to stop the progress of the disease, and this effort should be watched, and seconded by art. When the constitution is strengthened by good air, diet, and attention to the rules of Hygiene, the disease will not only stop, but the crooked bones will become straight, even without the aid of instruments. Of this Mr. Wilson has witnessed several instances. Our author has, for many years, been in the habit of demonstrating in his lectures, the wisdom of Nature in depositing abundance of osseous matter, when the bones begin to recover from the disease, at the part where it it most wanted—viz. on the inner part of the concave surface of their curve.

"Dr. Wm. Hunter, whose experience in the diseases of children was most extensive, recommended in rickets the constant use of those means which tend to strengthen the constitution: and he asserted that cold bathing corrected this habit more than any other remedy yet known, indeed that rickets, almost with certainty, could be prevented by the use of cold water; sea water, if the patient was near the sea, if not, spring water, the temperature of this being more constant than that of river water, and the use of it generally bringing on a healthy glow. The children, he observed, should be dipped daily, and only once at each period; they should be dried quickly, and friction by coarse linen, or flannel, should be applied to their backs and limbs for a considerable time afterwards; that great attention should be

* It is asserted by many good authors, and among others by Barthez and Portal that there is a precocious development of the intellectual powers, independently of moral causes, but as a characteristic feature of rickets. The organs of sense, especially those of sight and hearing, have a much greater degree of energy of function than in other children, and the volume of the brain is proportionally large. The bones composing the cranium, being soft, easily give way to this premature development of the cerebral mass, and its intellectual functions, the precocious energy of the latter, however, being of short duration; for, when the disease has advanced, the child becomes gradually more and more stupid.

paid to their breathing pure air, of a regular temperature, to cleanliness, wholesome food, and the state of the digestive organs. Let me add, that I have seen this system persevered in in many cases of rickets in all with some, and in most with very decided advantage to the patient." 168.

In very young children, Mr. Wilson thinks that instruments should never be applied to the limbs. They cannot either be necessary or useful; while by their weight and by preventing exercise, they must tend to increase the general debility. Their application should be deferred till the bones of the trunk have attained some firmness, and till those of the limb are hard enough not to be injured by the weight and pressure.

Incurvations of the spine are of two kinds—one from rickets, in which case the bend is usually to one side:—the other from caries of the bodies of the vertebræ, when the bend, of course, is forwards. It is to the first species (lateral) that the following observations apply.

In a female constitution merely weak, and without scrofulous affection of the other bones, the habit of continuing in a particular leaning attitude will often bring on this inclination, and, in a rickety constitution, will certainly produce it.

"When a bend has once been established, the superincumbent weight is thrown upon that part now in an unfavourable form for bearing it, and this of course increases the curve. Whenever there is a tendency to deviate from the perpendicular, the curve will continue to increase, or an attempt to counteract it, by a curve in another part of the spine, and in the opposite direction, will take place. We thus find, that in rickets the spine is bent serpentinely and laterally, resembling the italic *ʃ*, and is not, as in caries of the vertebræ, bent suddenly and forwards; and we often meet with several of these lateral curves from the attempts successively made to support the weight more favourably by counteraction." 173.

As there is generally more than one curve, it is evident that the altered shape of the spine cannot be owing to the greater contraction of the muscles on one side; nor to a partial deficiency of bony matter, since a redundancy of it is pressed out on the weak side. "It is, therefore, thinks our author, a fair inference that the curve is produced by weight or pressure having been long or frequently applied to a particular surface of the bones, in constitutions where, although every part of the bones has an equal supply of the phosphate of lime, yet in all of them, that supply is less than in strong and healthy bones."

The affection of the vertebral column often begins after the pelvis is well and fully formed, and therefore when the latter is not likely to be altered in shape, unless some constant and artificial pressure is made on certain parts of it. This should be borne in mind in all cases of spinal distortions in females. Mr. Wilson feels confident that, if timely attended to, such incurvations may, in general, be removed or remedied without mechanical instruments being employed, or any other distressing and violent means being used; "and that by this timely attention, the lives of many mothers and children may be saved, in cases where either mother or child, or perhaps both, must otherwise perish."

Although curvatures of the spine may be readily seen, and easily felt, by passing the fingers along the spinous processes when the patient stands before us, yet if she bends forward, without external support, the lateral curvations will often disappear. If she is admonished to hold herself erect too, and makes the attempt, she can immediately straighten her spine, and retain it a few seconds in its proper form. When this is the case, we may entertain well-founded hopes of a perfect recovery.

"And when the spine cannot in either of the above attitudes be rendered perfectly straight, we may still entertain no doubt of being able to arrest the progress and ameliorate the effects of the disease, by calling the natural powers of the body into proper action." 177.

Here our intelligent author adverts to the wonderful and beautiful mechanism of the spine, and the strength and motion it possesses from the combination of bones and muscles. Large masses of the latter are placed between the spinous and transverse processes of the vertebræ and the angles of the ribs, for the purposes of supportiug the weight of the head and upper extremities, and of varying the position of the chest and rest of the trunk. It is well known that these muscles are equal in power on both sides, and equally employed to support the spine in the natural erect position of the body.

"It is by calling these muscles into regular and frequent action, that the spine is to be restored to its natural shape and form, in cases where from weakness or rickets that form has been departed from." 177.

Mr. Wilson then goes on to observe that many complicated, and some ingenious instruments have been invented for taking off the weight of the head and upper extremities from the spine; and were this the only thing they accomplished, they, in many cases, would prove useful; but they too often throw the weight upon other bones that may and do bend under it.

"In the application of all the instruments that I have seen which would admit of loco-motion to the body, the weight, although removed from the vertebral colnmn, has been thrown upon the pelvis; either professedly so, or in a way a little disguised; and as the bones of the pelvis are not so hard in such patients as in persons who have no rickety disposition, the upper part or spine of the ilium on each side on which the weight is generally made to rest, is bent forwards, in consequence of which the pubes and ilia, where these bones form the fore-part of the brim of the pelvis, bend inwards, and diminish the aperture of that cavity so much, that the head of a child cannot even enter it, which to be born naturally should pass gradually through it. The preparations which I now produce are frightful, but useful examples of this distortion of the bones of the pelvis." 178.

Mr. Wilson has examined very many cases of incurvated spines, in women who could not afford expensive instruments, and in these he has found the pelvis so perfectly well formed as to allow the birth of living children, even where the spinal incurvations had been considerable and of long standing. On the contrary, he has examined other cases, where the affluence of the parties has enabled them to procure instruments, and in all of these he found the bones of the pelvis irreparably injured by having yielded to the additional burthen thrown on them, where they were not calculated to bear much weight. Specimens of this destructive practice were here shewn to the auditors by Mr. Wilson.

Such instruments as are fixed to parts, not of the patient's body, as chairs, or the ceiling of the room, may prove useful by taking off the weight from the spine; and, on the same principle, the horizontal position is beneficial.

" When seated, the chair may have an upright back, but no elbows or arms to it, for these would form a partial rest, which might prevent the muscles of the spine from being called into equal action, and it is by this equal action that the cure is to be effected." 180.

Here Mr. Wilson candidly acknowledges his obligations to Mr. Grant of Bath, for the first hints of curing this affection of the spine by the regular and uniform action of the muscles belonging to it.

" In an accidental conversation, he informed me, that he had proposed to cure the lateral incurvation of the spine, by placing a weight on the head of the patient, on the principle of producing frequent and equal action of the vertebral muscles; but that he seldom could convince, either the mothers, or even the medical men whom he had met in consultation, that by this weight he should succeed in effecting his object. His practice immediately struck me as founded on just physiological principles, and I told him that I had then a favourable opportunity of beginning a trial of it. On that very day I began the trial, and the event in three weeks exceeded my most sanguine expectations of success. Several years have passed since my conversation with Mr. Grant, but I have tried the plan in very many instances during the last sixteen years, and in no one, where it was properly persevered in, have I found it to fail in preventing the further progress of the disease, and in many I have witnessed it effecting a perfect cure, at least so perfect that no deformity was perceived, nor inconvenience in other respects suffered." 181.

Mr. Wilson illustrates the principle of the treatment thus: "if, says he, a finger is held up, and bent a little, a weight being placed on its tip, either will bend it completely, or oblige it to straighten itself so as to enable it to bear the weight when applied to it perpendicularly." It is in this way that the spine being bent in one or more directions, when a weight is added to the head, it directly and almost instinctively, by the action of its muscles, straightens itself to bear that weight; and this action often renewed, and persevered in for a moderate time, will recover the spine from the bend that otherwise must have increased; or in the attempt to remove it by instruments applied to the pelvis, their weight, and that of the body, must have effected and distorted that part, the perfect shape of which is important, not merely to the symetry, but to the life of the mother, or to the existence of the child.

"The weight may be used in the manner following: a small footstool, covered with a flat cushion, being inverted, may be placed on the patient's head, the hollow between the feet of the stool will allow of some substance, varying between four and ten pounds in weight, for it may be necessary to increase it to the last amount although much less is generally sufficient, to be placed in it; the patient should be instructed to raise this with both her arms, and support it on the crown of her head, elevating the spine at the same time towards the stool while held over her head; she then, preserving the most erect attitude she can, should walk in a straight line, as soldiers are taught to march, and for a time not exceeding ten minutes; this should be repeated occasionally during the day. By degrees she will learn to balance the weight, and this occasional exertion, giving the muscles their true action, will straighten the spine much more effectually and sooner than any mechanical instruments.

"The patient should be frequently reminded by her attendants to sit upright, and the momentary attempt to do this, even if the attitude cannot be long persevered in, will prove useful in forwarding the recovery. Negroe women and basket women, who early in life have been accustomed to carry heavy burthens on their heads, are never crooked." 183.

Various other modes of exercising the chest and limbs, in a regular, equal, and natural manner, will readily suggest themselves to every medical practitioner who has studied the formation of the skeleton, and the action of the principal muscles which are affixed to it, and move its several parts upon each other. The use of the cold and shower bath will aid the effect of the above exercise.

"In children a large towel dipped in cold spring water, and allowed to fall from the top of the back part of the head to the lower part of the trunk will answer as a good substitute for the bath. The patient should be immediately afterwards well dried, and gentle friction used for some little time on the skin, in the direction of the spine. The internal use of the preparations of steel are often serviceable. So far as my own experience goes, I have found the ferrum ammoniatum on the whole more useful than any other preparation of that substance." 187.

Having now presented our readers with the analysis of a single lecture, as a specimen of the whole, we have little doubt but that the favourable impression which it is calculated to make on unbiassed minds, will induce them to consult the original, and place it among the more valuable depositaries of surgical science for subsequent reference. In respect to the able and worthy author, we can add nothing to what we have said on a former occasion.

